# Citicoline: A Candidate for Adjunct Treatment of Multiple Sclerosis

**DOI:** 10.3390/ph14040326

**Published:** 2021-04-02

**Authors:** Paweł Grieb, Maciej Świątkiewicz, Agnieszka Kamińska, Anselm Jünemann, Robert Rejdak, Konrad Rejdak

**Affiliations:** 1 Department of Experimental Pharmacology, Mossakowski Medical Research Institute, Polish Academy of Sciences, 02-106 Warsaw, Poland; pgrieb@imdik.pan.pl; 2 Faculty of Medical Sciences, Collegium Medicum, Cardinal Stefan Wyszynski University, 01-938 Warsaw, Poland; a.kaminska@uksw.edu.pl; 3 Chair and Department of General and Pediatric Ophthalmology, Medical University of Lublin, 20-079 Lublin, Poland; anselm.juenemann@med.uni-rostock.de (A.J.); robert.rejdak@umlub.pl (R.R.); 4 Department of Neurology, Medical University of Lublin, 20-954 Lublin, Poland; konrad.rejdak@umlub.pl

**Keywords:** citicoline, multiple sclerosis, demyelination, remyelination

## Abstract

In remitting–relapsing multiple sclerosis (RR-MS), relapses are driven by autoreactive immune cells that enter the brain and spinal cord and damage myelin sheaths of axons in white and grey matter, whereas during remissions myelin is repaired by activated oligodendroglial cells. Disease-modifying therapies (DMTs) may either retard/attenuate myelin damage or promote/enhance/speed up myelin repair. Almost all currently approved DMTs inhibit myelin damage and are considerably toxic. Enhancement of myelin repair is considered an unmet medical need of MS patients. Citicoline, known for many years as a nootropic and neuroprotective drug and recently pronounced food supplement, has been found to be significantly efficacious in two complementary rodent models of MS, experimental autoimmune encephalomyelitis (EAE) and cuprizone-induced myelin toxicity. Moreover, citicoline treatment improves visual evoked potentials (VEPs) in glaucoma patients, which is relevant because VEP monitoring is frequently used as an indicator of remyelination in MS. Although over-the-counter availability of citicoline may impede its formal translation to the clinic of MS, evaluation of its efficacy for supporting remyelination in this disease is strongly indicated.

## 1. Introduction

Multiple sclerosis (MS) is a chronic inflammatory and demyelinating disease of the central nervous system (CNS), affecting between 2 and 2.5 million people throughout the world, mostly in the Northern Hemisphere [1]. Compared with the general healthy population, MS patients encounter significantly increased mortality, with life expectancy reduced by 7–14 years [2]. The disease is described as a brain- and spinal cord-specific chronic immunoaggressive process that affects the CNS, initially damaging myelin sheaths of axons [3].

**Figure 1:**
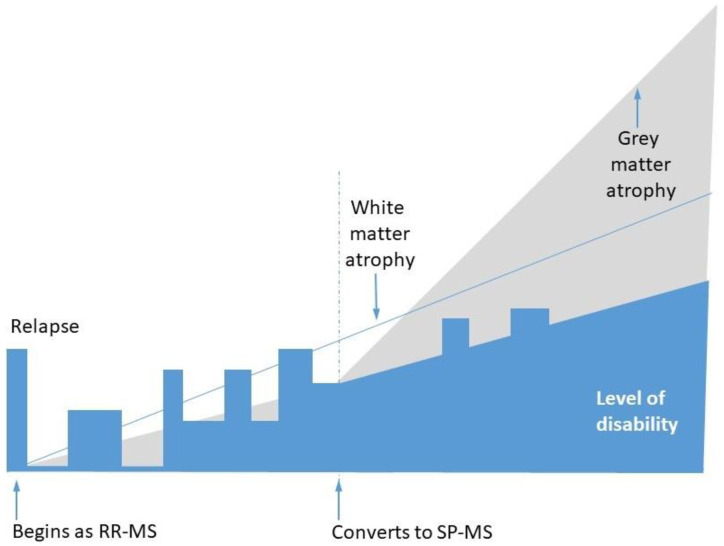
The most frequently encountered pattern of multiple sclerosis progression, including transition from relapsing–remitting multiple sclerosis (RR-MS) to secondary progressive multiple sclerosis (SP-MS) course. The thin blue line represents white matter (WM) atrophy, while the grey area depicts grey matter (GM) atrophy.

The clinical trajectory of MS in individual patients is variable; however, the most common pattern is as follows (Figure 1): the disease starts with a flare, frequently manifesting as optic neuritis, which later remits. In the months and years to come, it relapses and remits; however, after a few years, the relapses become incomplete, resulting in disability progression. This initial phase is called remitting–relapsing multiple sclerosis (RR-MS). Later, the disease changes to secondary progressive multiple sclerosis (SP-MS). In the transition period from RR-MS to SP-MS, relapses and remissions become less and less frequent, but neurological disability outside relapses continuously accumulates [4]. It is not clear whether RR-MS and SP-MS are two forms of the same disease or whether they represent distinct pathologies. Some patients suffering from RR-MS do not develop a secondary progressive course, whereas a small subgroup of patients from the beginning of the disease do not encounter relapses and remissions but develop continuous disability progression (this form is called Primary Progressive Multiple Sclerosis, PP-MS) [5].

Although in the last three decades several disease-modifying therapies for MS have been approved, there is a continuous need for further improvement. Available drugs are effective in controlling the inflammatory aspects of the disease, but supporting myelin regeneration is still an unmet medical need [6]. In the present essay we will overview current concepts concerning pathomechanisms and therapies of MS and justify the opinion that citicoline should be developed as an adjunct to pharmacological treatments of this disease.

## 2. Demyelination, Remyelination, and Neurodegeneration in MS

Myelin in the CNS is produced by oligodendrocytes. Myelin sheaths, wrapped around axons, enable rapid saltatory conduction of action potentials and contribute to the maintenance of axonal integrity.

Rodent and human CNS myelin is characterized by a high lipid content, its dry mass consisting of 70–85% lipids and 15–30% proteins. Circa 45% of these lipids are phospholipids, mainly phosphatidylethanolamine (PtdEth) and phosphatidylcholine (PtdCho) [7]. Studies on animals showed a very slow turnover rate of CNS myelin proteins [8], whereas turnover of lipids was not uniform, that of phospholipids being much faster than that of cholesterol (half-replacement times in adult mice were estimated as 20–25 days and 359 days, respectively) [9].

**Figure 2:**
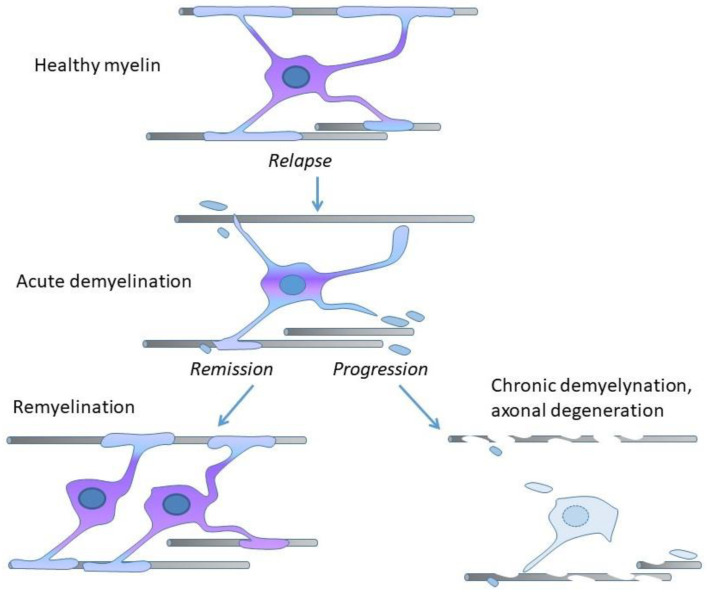
Central nervous system (CNS) axons are myelinated by oligodendrocytes (upper part: Healthy myelin). In RR-MS, during relapse, oligodendrocytes and myelin sheaths are destroyed (middle part: Acute demyelination). In disease remission the new oligodendrocytes generated from a widespread population of oligodendrocyte precursors put new myelin sheaths around the demyelinated axons (lower part, left side: Remyelination). Disease progression occurs when remyelination fails (lower part, right side: Chronic demyelination, axonal degeneration).

Demyelination is the process of or state resulting from the loss or destruction of myelin, whereas remyelination is the process of myelin restoration. In RR-MS, relapses are caused by immune cells invading CNS and damaging myelin, while remissions involve activation of oligodendroglia and oligodendroglial precursors, and remyelination, which protects axons from degeneration (Figure 2) [10].

In the background of the clinical evolution of MS a neurodegenerative process takes place, leading to the progressive atrophy of both white matter and grey matter. The most characteristic brain tissue injury in MS is demyelination with partial preservation of axons, while the other prominent pathological feature is brain atrophy [11]. Pathomechanisms responsible for these developments are the subject of continuous debate. According to a long-standing concept, currently described as the “outside-in” hypothesis, disease is initiated by infiltration of the brain and spinal cord with autoreactive lymphocytes and monocytes directed toward myelin sheaths of axons in white and grey matter. The results of some recent studies are, however, consistent with the alternative “inside-out” hypothesis according to which MS autoimmunity occurs subsequently to the primary CNS cytodegeneration affecting myelin sheaths [12].

Some data suggest that the pathological mechanisms responsible for brain tissues destruction differ quantitatively in RR-MS and SP-MS. On the other hand, in a pivotal paper by Bramow et al. [13] evidence was presented that, in progressive forms of MS, slowly expanding demyelination irreversibly destroys normal and repaired myelin. Thus, the degree of demyelination may be an important pathological correlate of clinical progression.

## 3. Preclinical Models of MS

Although animal models of human diseases never fully recapitulate their human prototypes, they are widely used for studies of the pathomechanism of diseases being modeled. According to a recent review, rodent models of MS may be classified as those designed to imitate either “outside-in” or “inside-out” pathomechanisms. Since the etiology of the disease is unknown, the use of both types of models is advised for the preclinical assessment of potential drugs [14].

The most popular “outside-in” model of MS is experimental autoimmune encephalomyelitis (EAE), leading to demyelination. EAE is evoked by immunization with CNS antigens, frequently supplemented with immune reaction enhancers, such as Freund’s adjuvant. At first glance, it seems to be adequate for evaluating drugs aimed at retarding or attenuating demyelination. The problem is that EAE is not a single model but a large family of models, each of them having somewhat different merits and different degrees of similarity with particular forms of MS [15]. Notwithstanding particulars, for the preclinical assessment of a drug, an EAE model is usually used in the following way: susceptible animals are immunized toward myelin antigen(s). Treatment is applied either in a preventive way, i.e., starting before signs of a disease become apparent, or as a therapeutic measure, i.e., starting when “clinical” symptoms appear. No standard has been established for measuring the effects of therapy; therefore, investigators define and use their own end points [16,17]. Considering the aforementioned diversity of EAE models, not surprising is their limited predictive value toward the clinical success of the therapies tested [18]. Nonetheless, many treatments (e.g., interferons beta, glatiramer) have shown benefit in EAE prior to being successfully tested in RR-MS, and the positive effect of such evaluation can be taken as a sign of increased probability of success in a clinical study.

The most popular “inside-out” model of MS, which may be particularly useful for testing therapies aimed at promoting, enhancing, and/or speeding up remyelination, is the cuprizone model. Cuprizone is a copper chelator, known for more than 50 years of its various toxicities, including demyelination which is a result of apoptosis of oligodendrocytes. The cuprizone model also is not a single standardized protocol, but a family of different paradigms executed on mice, rats, guinea pigs or hamsters. These models seem to lack the immune component of RR-MS but may be well suited for assessing remyelination, which develops after cessation of exposure to the toxin and involves a sequence of tightly orchestrated events that include limited inflammation, activation of microglia and astrocytes, and early recruitment of oligodendrocyte precursors [19].

## 4. Disease-Modifying Therapies for MS

In RR-MS, pharmacological therapies are expected to reduce the yearly incidence and/or decrease the length and severity of relapses, halt or slow down disability progression, and prevent or delay transition to SP-MS. Efficient treatments, called disease-modifying therapies (DMTs), may be classified as those that negatively alter (retard or attenuate) demyelination and those that positively alter (promote, enhance, and/or speed up) remyelination. Currently, 18 DMTs have been approved [20], almost all of which belong to the first group. They negatively alter demyelination during relapses of RR-MS through interfering with the patient’s immune system [21], and may further be classified as immunosuppressants which more or less indiscriminately suppress the activity of the immune system, and immunomodulators which act in a more selective way.

Although different drugs alter the function of the immune system differently, their common feature is posing the threat of adverse effects. The application of immunosuppressants is associated with an increased risk of infections [22]. Other types of adverse effects are more specifically related to particular drugs. For example, interferons beta may produce depression, thrombotic microangiopathy, hepatotoxicity, and flu-like syndromes; natalizumab, fingolimod, and dimethyl fumarate may cause progressive multifocal leukoencephalopathy; alemtuzumab may cause idiopathic thrombocytopenic purpura, autoimmune thyroid disease, thyroid cancer, etc. [23].

The second group of DMTs for RR-MS comprises drugs expected to promote, enhance, and speed up remyelination. Myelin maintenance and regulation is a dynamic process, and the description of its regulatory pathways paved the way to the identification of possible targets for therapeutic interventions. A promising new target is LINGO-1 (leucine-rich repeat and immunoglobulin-like domain-containing nogo receptor interacting protein 1), a negative regulator of oligodendrocyte differentiation [24]. Some drugs used in the treatment of other diseases have been suggested for repurposing to support remyelination. Enhancement of remyelination could also be achieved by securing availability of necessary raw materials. Recently, it has been suggested that supplying squalene, a precursor for the synthesis of cholesterol, may boost the repair of demyelinated lesions and have potential as a new strategy for MS treatment [25].

## 5. Visual Evoked Potentials for Monitoring Remyelination

Demyelinating lesions and axonal dysfunction lead to variable clinical syndromes, but optic nerve inflammation and/or lesions in other parts of the visual pathway occur in the majority of MS patients; the function of visual pathway may be employed as an indicator of brain damage [26]. Already in the early 1970s, delays in the visual evoked potentials (VEPs) were noted in patients suffering from optic neuritis, and the VEP technique was recommended as a supportive diagnostic tool. Currently, in MS diagnosis, VEPs have been replaced by magnetic resonance imaging and detection of oligoclonal bands in cerebrospinal fluid (CSF), but the former technique is still considered useful for monitoring disease progression [27]. An important aspect of VEPs (and other methods used to assess the structure and function of visual pathway, e.g., optical coherence tomography) in MS is their applicability as a translatable biomarker to track demyelination and functional remyelination in both preclinical research [28,29] and clinical trials [30,31].

The use of serial VEP recordings for therapy monitoring in MS was attempted more than three decades ago, but at that time there was no effective treatment, and the technique was found arduous [32]. More recently, VEP monitoring was employed in drug trials assessing patients with optic neuritis. Neuroprotective effects of such drugs as erythropoietin [33], simvastatin [34], phenytoin [35], and anti-LINGO-1 antibody opicinumab [36] were evaluated with this technique. In another recent study of patients with acute unilateral optic neuritis, a significant shortening of VEP latency after six months of fingolimod treatment was reported [37].

## 6. Pharmacodynamics of Citicoline

Citicoline is cytidine diphosphocholine (CDP-choline) from an exogenous source. CDP-choline of endogenous origin is a natural constituent of all living cells, which serves as a critical metabolite in the pathway of de novo synthesis of phosphatidylcholine (PCho). Intake of citicoline enhances memory in both rodents [38] and humans [39]. In the Anatomical Therapeutic Chemical (ATC) classification of drugs, citicoline is listed in the subgroup “Other psychostimulants and nootropics”, under the code N06BX06. (The term “nootropic” was proposed in 1972 to describe drugs; supplements; and other substances that improve cognitive functions, such as memory, motivation, and creativity [40]). The positive effects of citicoline on cognition have been related to increases in some brain neurotransmitters [41].

Besides nootropic properties, citicoline has repeatedly shown to provide significant neuroprotection in various preclinical models of brain ischemia and trauma. It was devoid of toxicity and seemed to help neurons survive insults. Unfortunately, two large pivotal trials failed to confirm its clinical efficacy in either ischemic stroke or brain trauma. The likely reason was that the severity of acute neurodegenerative processes overwhelmed any treatment effect. There is also some controversy related to the equivalence of injectable and oral formulations [42].

While clinically not effective in acute brain disorders that involve rapid and massive neuronal death, citicoline seems to provide benefits in slowly developing neurodegenerative diseases, such as degeneration of neurons that underlies memory loss in mild vascular cognitive impairment [39], and retinal ganglion cells and optic nerve degeneration in primary open-angle glaucoma [43]. In the recent review on citicoline in the context of glaucoma [44], multiple mechanisms of action of this compound are listed that may mediate its neuroprotective, neurorestorative, and neuroregenerative properties. Some of these, namely, preservation of cardiolipin, sphingomyelin and arachidonic acid content of PtdCho and PtdEth, and restoration of PtdCho, are certainly pertinent to myelin synthesis and remyelination.

## 7. Assessment of Citicoline in Preclinical Models of MS

In a pivotal study published online at the end of 2004, German investigators [45] presented evidence for the therapeutic efficacy of citicoline in two complementary murine models of MS, namely, EAE and cuprizone intoxication. EAE was induced by myelin oligodendrocyte glycoprotein (MOG) immunization. Toxic demyelination was induced by feeding mice a diet containing 0.2% cuprizone. Neurological status of the animals was assessed daily and expressed in a 5-point scale. These data were supplemented by an extensive battery of behavioral, histological, immunohistochemical, electron microscopical, cellular, and molecular biology tests aimed at dissecting mechanisms of citicoline action.

**Figure 3:**
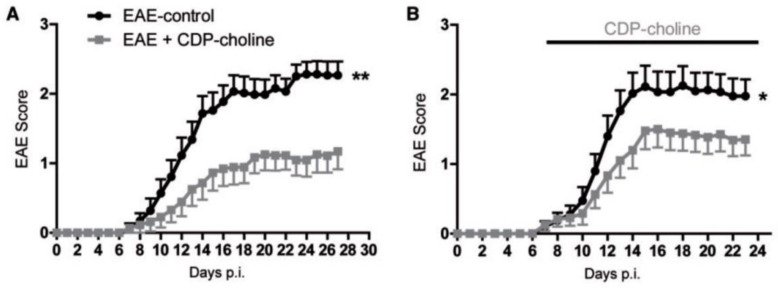
Citicoline (CDP-choline) ameliorated neurological symptoms of experimental autoimmune encephalomyelitis (EAE) in rats. Panel (**A**), preventive treatment; panel (**B**), treatment initiated at symptoms onset. Days p.i. means days after inoculation with MOG. Statistical significance: * *p* < 0.05, ** *p* < 0.01. Reproduced from [45], by permission of Oxford University Press.

In the EAE part of the study, there were three active treatment groups receiving 500 mg/kg citicoline daily by oral gavage, repeated till the end of the experiment: One group included mice treated in a preventive way, i.e., starting on the day of immunization; mice in the other groups were treated therapeutically, starting on a day when neurological symptoms were detected, or one week later. Vehicle-treated animals served as controls. The major findings were as follows: (i) Citicoline was most effective when given preventively; treatment initiated when neurological symptoms were detected was less effective (Figure 3); and treatment initiated one week later was not effective at all. (ii) In citicoline-treated mice, beneficial neurological effects occurred without the attenuation of signs of neuroinflammation. (iii) On days 15 and 27 after the EAE induction, no change was found between the brains of citicoline-treated and control animals in quantities of CD3-positive CNS infiltrating T lymphocytes, in infiltrating macrophages/microglia, in the number of infiltrates, and in the extent of infiltrate area. (iv) Several tests, such as assessment of cytokine production and T cell proliferation following antigen restimulation in vitro, did not provide any evidence of modulation of myeloid cells and T cell responses by citicoline. (v) At the same time, in citicoline-treated animals, higher numbers of oligodendrocytes and signs of increased myelination were found.

**Figure 4:**
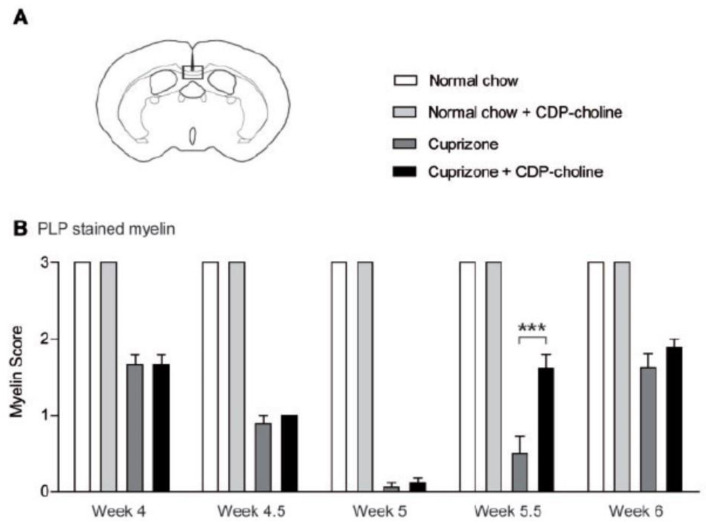
Citicoline (CDP-choline) accelerated remyelination in the corpus callosum of mice 0.5 weeks after cuprizone withdrawal. Mice were fed with cuprizone for five weeks to induce demyelination. Later, cuprizone was withdrawn, and mice were allowed to remyelinate. The extent of myelination was judged by scoring of proteolipid protein (PLP) in a blinded manner by three observers and expressed in a 3-point scale from 0 (complete demyelination) to 3 (normal myelin). In (**A**) the box marks the area of the brain coronal section in which myelin scoring was performed, shown in (**B**). Statistical significance: *** *p* < 0.001. Reproduced from [45], by permission of Oxford University Press.

In the cuprizone part of the study, the animals were treated with citicoline (500 mg/kg) or sham every day beginning on the day of cuprizone-feeding until termination of the experiment, or for shorter periods of time. Here, the major findings were as follows: (i) As expected, mice responded to five weeks of cuprizone treatment by showing marked demyelination of the corpus callosum. (ii) Citicoline treatment did not influence cuprizone-induced demyelination, but it markedly accelerated and enhanced remyelination when applied following cessation of cuprizone treatment (Figure 4), which was accompanied by an increased number of proliferating oligodendrocytes. (iii) Behavioral tests showed that remyelination enhanced by citicoline was effective in reversing demyelination-associated motor coordination impairment. Further in vitro experiments showed that citicoline does not modify the function of microglia and macrophages. The authors interpreted these findings as evidence for citicoline’s effectiveness by increasing the proliferation rate of oligodendrocyte precursor cells, resulting in more mature oligodendrocytes.

**Figure 5:**
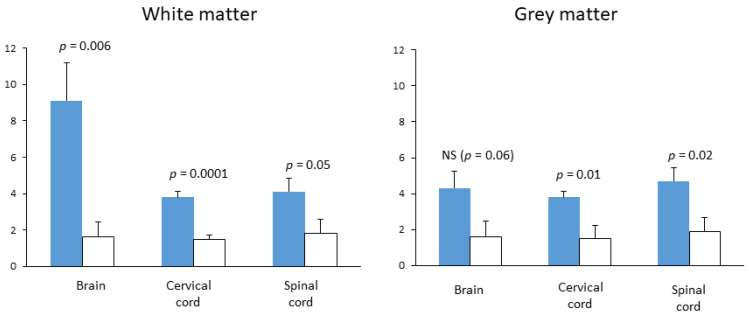
The effect of citicoline treatment on the average number of inflammatory infiltrates in brain and spinal cord transections of rats with EAE. Female Lewis rats were immunized by intradermal injection of guinea pig spinal cord homogenate (50%) with complete Freund’s adjuvant and Mycobacterium phlei (11 mg/mL); treatments with citicoline (500 mg/kg ip, qd) or vehicle (*n* = 7 per group) started on day 6; brain and spinal cord fixed by transcardiac perfusion on day 14 after immunization. Inflammatory infiltrates were counted under optical microscope on brain and spinal cord cross-sections stained with hematoxylin-eosin and cresyl violet stain; these data are presented as means and standard deviations. Statistical significance of differences evaluated by Mann–Whitney U test. (presented at the 56th meeting of American Academy of Neurology, San Francisco [48]).

In 2014, the aforementioned publication was pronounced the best basic science paper in *Multiple Sclerosis* [46], and the authors certainly deserved this applause. They employed a large number of techniques and tests and tried to present a cohesive interpretation of their findings. Moreover, their neurological findings were corroborated by two studies published previously, but only in the abstract form. The first study [47], which was industry-sponsored, showed the ability of citicoline to attenuate the development of EAE induced in rats and mice by injection of heterologous spinal cord and myelin basic protein (both in complete Freund’s adjuvant), respectively. The second one [48] was conducted in our laboratory and was not industry-sponsored (it was presented as a poster at the 56th meeting of the American Academy of Neurology in San Francisco, but it did not gain attention, which unfortunately discouraged us from submitting the full report). The aim of our exercise was to test whether daily intraperitoneal injections of citicoline starting at day 7 after inoculation influence the intensity of CNS inflammation in EAE. Treatment with citicoline significantly attenuated signs of inflammatory process, particularly in the brain and cervical cord white matter (Figure 5).

## 8. Citicoline Should Be Evaluated in MS

Although the pivotal study on the efficacy of citicoline in preclinical models of MS has been applauded, it has not had any bearing on MS therapy. Virtually none of the dozens of reviews dedicated to current and emerging treatments for this disease even mentions citicoline. One exception is a “state of the art” review devoted to the issue of remyelination as a new treatment strategy to fight CNS diseases [49]. In this article, published shortly after the publication of the aforementioned pivotal study, 21 potential drugs related to myelin repair therapies were listed, divided into three categories: those already in use for the other indications, new drugs already in clinical trials for MS and experimental compounds that in preclinical evaluation showed signs of pro-remyelinating activity. Citicoline was, indeed, listed in the “already in use” category, but no further information was provided. Instead, it was mentioned that it failed in studies investigating cerebral ischemia, which does not look to be a good recommendation, and that to date, no clinical studies of its efficacy in MS are underway. In another review [6], citicoline was mentioned marginally under the heading “Miscellaneous and other medicines”.

In another commentary on new directions in remyelination research [50], citicoline was also not mentioned; however, the author remarked that one leading candidate for a surrogate clinical end point to track remyelination is visual evoked potentials (VEP). In this context, it is certainly worth to mention that the principal application of VEP recordings is for diagnostics and therapy monitoring of primary open angle glaucoma (POAG), an ophthalmic condition currently considered a neurodegenerative disease [51]. In POAG patients, citicoline treatment, parenteral as well as oral, has been repeatedly shown to increase amplitude and decrease latency of VEPs [52,53,54]. Moreover, in POAG patients, oral citicoline treatment halted the decrease in retinal nerve fiber layer (RNFL) and ganglion cell complex (GCC), their thickness monitored by optical coherence tomography (OCT) [55]. Interestingly, many MS patients have thinner retinal layers than healthy controls, and retinal thickness measured with OCT correlates with their disability and risk of disability worsening [56,57].

Citicoline was recently pronounced a food ingredient in major world markets [58], and there is no known contraindication of its intake. When, in the treatment of patients suffering from Alzheimer’s disease, oral intake of this ingredient was added to the treatment with anticholinesterase inhibitors, positive clinical effects in the domains of cognition, mood, and behavioral symptoms were reported [59]. These effects may be related to the stimulation of phospholipid synthesis, resulting in stabilization of brain neuronal membranes [60], and to increased choline availability for synthesis of acetylcholine [61], but also to the improvement of myelination.

The significance of the last mechanism is supported by a recent study describing the changes in the corpus callosum diffusion tensor imaging (DTI) parameters in patients with leukoaraiosis (LA) following one year of oral citicoline treatment [62]. The term leukoaraiosis was introduced in 1986 by Canadian radiologists to describe areas of the white matter with decreased density in brain CT scans and changed signals in brain magnetic resonance images, detected in old patients and/or patients suffering from various neurological diseases, including MS [63]. LA is thought to reflect damage to the axons and myelin in the corpus callosum, secondary either to cerebral small vessel disease or the degeneration of brain white matter, and associated with cognitive decline [64]. The aforementioned study [62] showed that, compared to untreated controls, patients taking citicoline displayed significantly smaller changes or even signs of improvement in fractional anisotropy (FA) and mean diffusivity (MD), the DTI parameters known to be roughly related to the myelin content [65].

In the context of MS, particularly the progressive forms of this disease, it is worth to note that myelin is not only a major component of the white matter but is also present in grey matter [66], although in much smaller quantities. In multiple metabolomic studies, increased levels of CSF choline and altered plasma levels of certain phospholipids, particularly those containing choline, were detected in MS patients [67,68,69,70]. Metabolomic studies cannot identify the source of these alterations. However, the in vivo proton magnetic resonance spectroscopy (^1^H-MRS) studies indicate that phospholipids not associated with myelin are not significantly affected in MS. ^1^H-MRS enables one to quantify cytosolic “NMR-visible” choline compounds in the brain in vivo as the so-called “choline resonance signal”. It has repeatedly been shown that in the brains of MS patients, the increased choline resonance signal is recorded from plaques, whereas the signals from either normally appeared white matter or grey matter are not cohesive (see [71] and the references cited).

We conclude that citicoline certainly deserves attention as a food substance that, when added to the current treatment regime of RR-MS, may enhance and accelerate remyelination. Its possible benefit for patients suffering from progressive forms of MS may also deserve evaluation.

## Data Availability

No new data were created or analyzed in this study. Data sharing is not applicable to this article.
